# Supplementary material for: A fault-intrusion-tolerant system and deadline-aware algorithm for scheduling scientific workflow in the cloud
Source: PeerJ Comput Sci. 2021 Nov 2;7:e747. doi: 10.7717/peerj-cs.747 (PMC8576554; doi:10.7717/peerj-cs.747)
Supplement: Supplemental Information 6 [file peerj-cs-07-747-s006.zip › Myproject1.1/docs/index-files/index-12.html]

M-Index


JavaScript is disabled on your browser.


- Overview
- Package
- Class
- Use
- Tree
- Deprecated
- Index
- Help

- Prev Letter
- Next Letter

- Frames
- No Frames

- All Classes

A B C D E F G H I J L M N O P Q R S T U V W Z 


## M

mad(double[]) - Static method in class org.cloudbus.cloudsim.util.MathUtil
:   Gets the mad.

MadMc - Class in org.cloudbus.cloudsim.examples.power.planetlab
:   A simulation of a heterogeneous power aware data center that applies the Median Absolute
    Deviation (MAD) VM allocation policy and Maximum Correlation (MC) VM selection policy.

MadMc() - Constructor for class org.cloudbus.cloudsim.examples.power.planetlab.MadMc


MadMc - Class in org.cloudbus.cloudsim.examples.power.random
:   A simulation of a heterogeneous power aware data center that applies the Median Absolute
    Deviation (MAD) VM allocation policy and Maximum Correlation (MC) VM selection policy.

MadMc() - Constructor for class org.cloudbus.cloudsim.examples.power.random.MadMc


MadMmt - Class in org.cloudbus.cloudsim.examples.power.planetlab
:   A simulation of a heterogeneous power aware data center that applies the Median Absolute
    Deviation (MAD) VM allocation policy and Minimum Migration Time (MMT) VM selection policy.

MadMmt() - Constructor for class org.cloudbus.cloudsim.examples.power.planetlab.MadMmt


MadMmt - Class in org.cloudbus.cloudsim.examples.power.random
:   A simulation of a heterogeneous power aware data center that applies the Median Absolute
    Deviation (MAD) VM allocation policy and Minimum Migration Time (MMT) VM selection policy.

MadMmt() - Constructor for class org.cloudbus.cloudsim.examples.power.random.MadMmt


MadMu - Class in org.cloudbus.cloudsim.examples.power.planetlab
:   A simulation of a heterogeneous power aware data center that applies the Median Absolute
    Deviation (MAD) VM allocation policy and Minimum Utilization (MU) VM selection policy.

MadMu() - Constructor for class org.cloudbus.cloudsim.examples.power.planetlab.MadMu


MadMu - Class in org.cloudbus.cloudsim.examples.power.random
:   A simulation of a heterogeneous power aware data center that applies the Median Absolute
    Deviation (MAD) VM allocation policy and Minimum Utilization (MU) VM selection policy.

MadMu() - Constructor for class org.cloudbus.cloudsim.examples.power.random.MadMu


MadRs - Class in org.cloudbus.cloudsim.examples.power.planetlab
:   A simulation of a heterogeneous power aware data center that applies the Median Absolute
    Deviation (MAD) VM allocation policy and Random Selection (RS) VM selection policy.

MadRs() - Constructor for class org.cloudbus.cloudsim.examples.power.planetlab.MadRs


MadRs - Class in org.cloudbus.cloudsim.examples.power.random
:   A simulation of a heterogeneous power aware data center that applies the Median Absolute
    Deviation (MAD) VM allocation policy and Random Selection (RS) VM selection policy.

MadRs() - Constructor for class org.cloudbus.cloudsim.examples.power.random.MadRs


main(String[]) - Static method in class org.cloudbus.cloudsim.examples.CloudSimExample1
:   Creates main() to run this example.

main(String[]) - Static method in class org.cloudbus.cloudsim.examples.CloudSimExample2
:   Creates main() to run this example

main(String[]) - Static method in class org.cloudbus.cloudsim.examples.CloudSimExample3
:   Creates main() to run this example

main(String[]) - Static method in class org.cloudbus.cloudsim.examples.CloudSimExample4
:   Creates main() to run this example

main(String[]) - Static method in class org.cloudbus.cloudsim.examples.CloudSimExample5
:   Creates main() to run this example

main(String[]) - Static method in class org.cloudbus.cloudsim.examples.CloudSimExample6
:   Creates main() to run this example

main(String[]) - Static method in class org.cloudbus.cloudsim.examples.CloudSimExample7
:   Creates main() to run this example

main(String[]) - Static method in class org.cloudbus.cloudsim.examples.CloudSimExample8
:   Creates main() to run this example

main(String[]) - Static method in class org.cloudbus.cloudsim.examples.network.datacenter.TestExample
:   Creates main() to run this example.

main(String[]) - Static method in class org.cloudbus.cloudsim.examples.network.NetworkExample1
:   Creates main() to run this example

main(String[]) - Static method in class org.cloudbus.cloudsim.examples.network.NetworkExample2
:   Creates main() to run this example

main(String[]) - Static method in class org.cloudbus.cloudsim.examples.network.NetworkExample3
:   Creates main() to run this example

main(String[]) - Static method in class org.cloudbus.cloudsim.examples.network.NetworkExample4
:   Creates main() to run this example

main(String[]) - Static method in class org.cloudbus.cloudsim.examples.power.planetlab.Dvfs
:   The main method.

main(String[]) - Static method in class org.cloudbus.cloudsim.examples.power.planetlab.IqrMc
:   The main method.

main(String[]) - Static method in class org.cloudbus.cloudsim.examples.power.planetlab.IqrMmt
:   The main method.

main(String[]) - Static method in class org.cloudbus.cloudsim.examples.power.planetlab.IqrMu
:   The main method.

main(String[]) - Static method in class org.cloudbus.cloudsim.examples.power.planetlab.IqrRs
:   The main method.

main(String[]) - Static method in class org.cloudbus.cloudsim.examples.power.planetlab.LrMc
:   The main method.

main(String[]) - Static method in class org.cloudbus.cloudsim.examples.power.planetlab.LrMmt
:   The main method.

main(String[]) - Static method in class org.cloudbus.cloudsim.examples.power.planetlab.LrMu
:   The main method.

main(String[]) - Static method in class org.cloudbus.cloudsim.examples.power.planetlab.LrrMc
:   The main method.

main(String[]) - Static method in class org.cloudbus.cloudsim.examples.power.planetlab.LrrMmt
:   The main method.

main(String[]) - Static method in class org.cloudbus.cloudsim.examples.power.planetlab.LrrMu
:   The main method.

main(String[]) - Static method in class org.cloudbus.cloudsim.examples.power.planetlab.LrrRs
:   The main method.

main(String[]) - Static method in class org.cloudbus.cloudsim.examples.power.planetlab.LrRs
:   The main method.

main(String[]) - Static method in class org.cloudbus.cloudsim.examples.power.planetlab.MadMc
:   The main method.

main(String[]) - Static method in class org.cloudbus.cloudsim.examples.power.planetlab.MadMmt
:   The main method.

main(String[]) - Static method in class org.cloudbus.cloudsim.examples.power.planetlab.MadMu
:   The main method.

main(String[]) - Static method in class org.cloudbus.cloudsim.examples.power.planetlab.MadRs
:   The main method.

main(String[]) - Static method in class org.cloudbus.cloudsim.examples.power.planetlab.NonPowerAware
:   The main method.

main(String[]) - Static method in class org.cloudbus.cloudsim.examples.power.planetlab.PlanetLabRunnerConsole
:   The main method.

main(String[]) - Static method in class org.cloudbus.cloudsim.examples.power.planetlab.ThrMc
:   The main method.

main(String[]) - Static method in class org.cloudbus.cloudsim.examples.power.planetlab.ThrMmt
:   The main method.

main(String[]) - Static method in class org.cloudbus.cloudsim.examples.power.planetlab.ThrMu
:   The main method.

main(String[]) - Static method in class org.cloudbus.cloudsim.examples.power.planetlab.ThrRs
:   The main method.

main(String[]) - Static method in class org.cloudbus.cloudsim.examples.power.random.Dvfs
:   The main method.

main(String[]) - Static method in class org.cloudbus.cloudsim.examples.power.random.IqrMc
:   The main method.

main(String[]) - Static method in class org.cloudbus.cloudsim.examples.power.random.IqrMmt
:   The main method.

main(String[]) - Static method in class org.cloudbus.cloudsim.examples.power.random.IqrMu
:   The main method.

main(String[]) - Static method in class org.cloudbus.cloudsim.examples.power.random.IqrRs
:   The main method.

main(String[]) - Static method in class org.cloudbus.cloudsim.examples.power.random.LrMc
:   The main method.

main(String[]) - Static method in class org.cloudbus.cloudsim.examples.power.random.LrMmt
:   The main method.

main(String[]) - Static method in class org.cloudbus.cloudsim.examples.power.random.LrMu
:   The main method.

main(String[]) - Static method in class org.cloudbus.cloudsim.examples.power.random.LrrMc
:   The main method.

main(String[]) - Static method in class org.cloudbus.cloudsim.examples.power.random.LrrMmt
:   The main method.

main(String[]) - Static method in class org.cloudbus.cloudsim.examples.power.random.LrrMu
:   The main method.

main(String[]) - Static method in class org.cloudbus.cloudsim.examples.power.random.LrrRs
:   The main method.

main(String[]) - Static method in class org.cloudbus.cloudsim.examples.power.random.LrRs
:   The main method.

main(String[]) - Static method in class org.cloudbus.cloudsim.examples.power.random.MadMc
:   The main method.

main(String[]) - Static method in class org.cloudbus.cloudsim.examples.power.random.MadMmt
:   The main method.

main(String[]) - Static method in class org.cloudbus.cloudsim.examples.power.random.MadMu
:   The main method.

main(String[]) - Static method in class org.cloudbus.cloudsim.examples.power.random.MadRs
:   The main method.

main(String[]) - Static method in class org.cloudbus.cloudsim.examples.power.random.NonPowerAware
:   Creates main() to run this example.

main(String[]) - Static method in class org.cloudbus.cloudsim.examples.power.random.ThrMc
:   The main method.

main(String[]) - Static method in class org.cloudbus.cloudsim.examples.power.random.ThrMmt
:   The main method.

main(String[]) - Static method in class org.cloudbus.cloudsim.examples.power.random.ThrMu
:   The main method.

main(String[]) - Static method in class org.cloudbus.cloudsim.examples.power.random.ThrRs
:   The main method.

main(String[]) - Static method in class org.workflowsim.examples.WorkflowSimExample1
:   Creates main() to run this example
    This example has only one datacenter and one storage

main(String[]) - Static method in class org.workflowsim.examples.WorkflowSimExample2
:   Creates main() to run this example

makeMasterCopy() - Method in class org.cloudbus.cloudsim.File
:   Clone this file and make the new file as a **master** copy as well

makeReplica() - Method in class org.cloudbus.cloudsim.File
:   Clone this file but the clone file is set to a **replica**

map - Static variable in class org.cloudbus.cloudsim.NetworkTopology


mapNode(int, int) - Static method in class org.cloudbus.cloudsim.NetworkTopology
:   Maps a CloudSim entity to a node in the network topology

match(SimEvent) - Method in class org.cloudbus.cloudsim.core.predicates.Predicate
:   The match function which must be overridden when writing a new predicate.

match(SimEvent) - Method in class org.cloudbus.cloudsim.core.predicates.PredicateAny
:   The match function called by `Simulation`, not used directly by the user.

match(SimEvent) - Method in class org.cloudbus.cloudsim.core.predicates.PredicateFrom
:   The match function called by `Simulation`, not used directly by the user.

match(SimEvent) - Method in class org.cloudbus.cloudsim.core.predicates.PredicateNone
:   The match function called by `Simulation`, not used directly by the user.

match(SimEvent) - Method in class org.cloudbus.cloudsim.core.predicates.PredicateNotFrom
:   The match function called by `Simulation`, not used directly by the user.

match(SimEvent) - Method in class org.cloudbus.cloudsim.core.predicates.PredicateNotType
:   The match function called by `Simulation`, not used directly by the user.

match(SimEvent) - Method in class org.cloudbus.cloudsim.core.predicates.PredicateType
:   The match function called by `Sim_system`, not used directly by the user.

MathUtil - Class in org.cloudbus.cloudsim.util
:   A class containing multiple convenient math functions.

MathUtil() - Constructor for class org.cloudbus.cloudsim.util.MathUtil


maxhostVM - Static variable in class org.cloudbus.cloudsim.network.datacenter.NetworkConstants


maxMemperVM - Static variable in class org.cloudbus.cloudsim.network.datacenter.NetworkConstants


MaxMinScheduler - Class in org.workflowsim.scheduler
:   MaxMin algorithm.

MaxMinScheduler() - Constructor for class org.workflowsim.scheduler.MaxMinScheduler
:   Initialize a MaxMin scheduler.

MCTScheduler - Class in org.workflowsim.scheduler
:   MCT algorithm

MCTScheduler() - Constructor for class org.workflowsim.scheduler.MCTScheduler


mDelayMatrix - Variable in class org.cloudbus.cloudsim.network.DelayMatrix\_Float
:   matrix holding delay information between any two nodes

mean(List<Double>) - Static method in class org.cloudbus.cloudsim.util.MathUtil
:   Gets the average.

median(List<Double>) - Static method in class org.cloudbus.cloudsim.util.MathUtil
:   Gets the median.

median(double[]) - Static method in class org.cloudbus.cloudsim.util.MathUtil
:   Gets the median.

memory - Variable in class org.cloudbus.cloudsim.network.datacenter.NetworkHost


memory - Variable in class org.cloudbus.cloudsim.network.datacenter.NetworkVm


migrateCloudlet() - Method in class org.cloudbus.cloudsim.CloudletScheduler
:   Returns one cloudlet to migrate to another vm.

migrateCloudlet() - Method in class org.cloudbus.cloudsim.CloudletSchedulerSpaceShared
:   Returns one cloudlet to migrate to another vm.

migrateCloudlet() - Method in class org.cloudbus.cloudsim.CloudletSchedulerTimeShared
:   Returns one cloudlet to migrate to another vm.

migrateCloudlet() - Method in class org.cloudbus.cloudsim.network.datacenter.NetworkCloudletSpaceSharedScheduler
:   Returns one cloudlet to migrate to another vm.

MILLION - Static variable in class org.cloudbus.cloudsim.Consts


MinMinScheduler - Class in org.workflowsim.scheduler
:   MinMin algorithm.

MinMinScheduler() - Constructor for class org.workflowsim.scheduler.MinMinScheduler


mName2Runtime - Variable in class org.workflowsim.WorkflowParser
:   Map from task name to task runtime.

mName2Size - Variable in class org.workflowsim.WorkflowParser
:   Map from file name (data) to its size.

mName2Task - Variable in class org.workflowsim.WorkflowParser
:   Map from task name to task.

move(List<T>, List<T>, T) - Static method in class org.cloudbus.cloudsim.lists.ResCloudletList
:   Move a ResCloudlet object from this linked-list into a specified one.

mTotalNodeNum - Variable in class org.cloudbus.cloudsim.network.DelayMatrix\_Float
:   number of nodes in the distance-aware-topology

A B C D E F G H I J L M N O P Q R S T U V W Z

- Overview
- Package
- Class
- Use
- Tree
- Deprecated
- Index
- Help

- Prev Letter
- Next Letter

- Frames
- No Frames

- All Classes
